# Genetics of diabetes-associated microvascular complications

**DOI:** 10.1007/s00125-023-05964-x

**Published:** 2023-07-14

**Authors:** Valeriya Lyssenko, Allan Vaag

**Affiliations:** 1 Department of Clinical Science, Mohn Research Center for Diabetes Precision Medicine, University of Bergen, Bergen, Norway; 2 Department of Clinical Sciences, Lund University Diabetes Center, Lund University, Lund, Sweden; 3 Steno Diabetes Center Copenhagen, Herlev, Denmark

**Keywords:** Diabetes, DNA, Genetics, Intrauterine programming, Metabolism, Microvascular diseases, Nephropathy, Neuropathy, Retinopathy, Review

## Abstract

Diabetes is associated with excess morbidity and mortality due to both micro- and macrovascular complications, as well as a range of non-classical comorbidities. Diabetes-associated microvascular complications are those considered most closely related to hyperglycaemia in a causal manner. However, some individuals with hyperglycaemia (even those with severe hyperglycaemia) do not develop microvascular diseases, which, together with evidence of co-occurrence of microvascular diseases in families, suggests a role for genetics. While genome-wide association studies (GWASs) produced firm evidence of multiple genetic variants underlying differential susceptibility to type 1 and type 2 diabetes, genetic determinants of microvascular complications are mostly suggestive. Identified susceptibility variants of diabetic kidney disease (DKD) in type 2 diabetes mirror variants underlying chronic kidney disease (CKD) in individuals without diabetes. As for retinopathy and neuropathy, reported risk variants currently lack large-scale replication. The reported associations between type 2 diabetes risk variants and microvascular complications may be explained by hyperglycaemia. More extensive phenotyping, along with adjustments for unmeasured confounding, including both early (fetal) and late-life (hyperglycaemia, hypertension, etc.) environmental factors, are urgently needed to understand the genetics of microvascular complications. Finally, genetic variants associated with reduced glycolysis, mitochondrial dysfunction and DNA damage and sustained cell regeneration may protect against microvascular complications, illustrating the utility of studies in individuals who have escaped these complications.

**Graphical Abstract:**

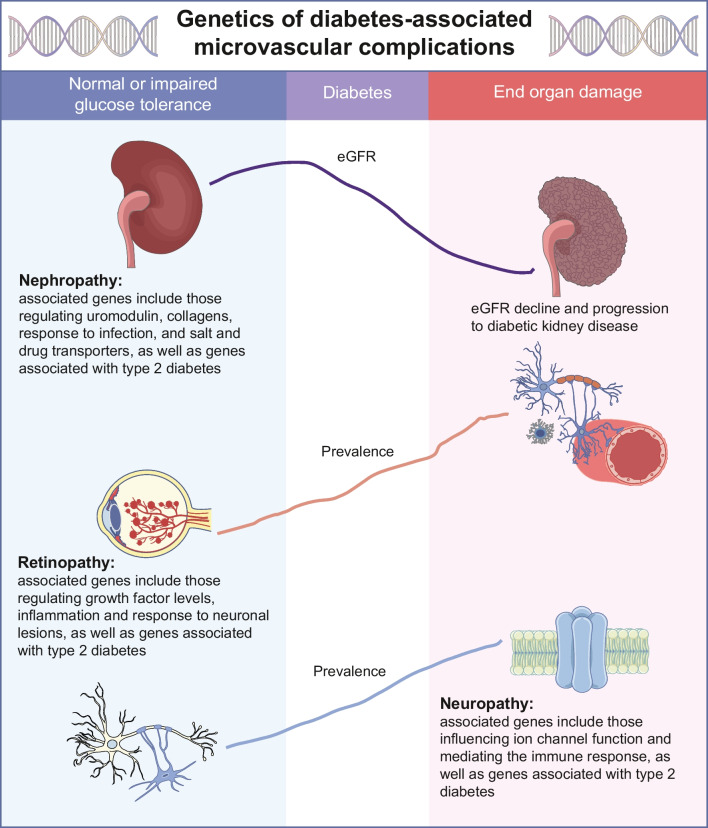

**Supplementary Information:**

The online version contains a slide of the figure for download available at 10.1007/s00125-023-05964-x.


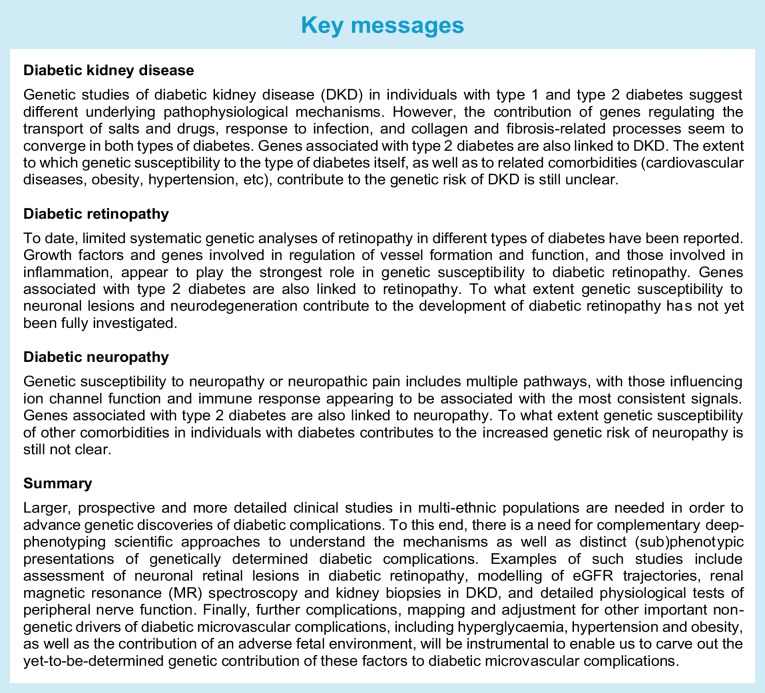


## Introduction

Diabetes is one of the leading causes of blindness, end-stage kidney failure, limb amputation, heart disease, stroke, cirrhosis of the liver and premature death. Diabetes is diagnosed by elevated glucose levels, which accurately reflects the core pathophysiology of pancreatic beta cell destruction in type 1 diabetes. In type 2 diabetes, hyperglycaemia occurs as the result of multiple organ dysfunction and is followed by more widespread dysmetabolic changes, including dyslipidaemia, subclinical inflammation and altered coagulation. Almost 25 years ago, the DCCT, conducted in individuals with type 1 diabetes, showed that chronically elevated levels of glucose were associated with increased risk of vascular complications [1]. Intensive glucose control has also been shown to reduce risk of microvascular complications in individuals with type 2 diabetes [2]. The sequence of events leading to organ damage in individuals with type 2 diabetes is not easy to delineate given the prolonged period of diabetes unawareness and the presence of signs of complications at the time of disease diagnosis [3]. Currently, the major vascular complications of diabetes are grouped into microvascular complications (damage of small vessels or neurofibres), comprising retinopathy, nephropathy and neuropathy, and macrovascular complications (damage of large vessels), comprising cardiovascular diseases such as myocardial infarction, stroke, peripheral arteriosclerotic disease, and can result in death [3]. In addition, there are other comorbidities, such as non-alcoholic fatty liver disease (NAFLD) [4], dementia, Alzheimer’s disease, cancer, infectious diseases and sleep apnoea, that frequently occur in individuals with diabetes [5].

Although the prevailing opinion is that chronic hyperglycaemia is a driving cause of complications in diabetes, clinical observations showed that individuals with similar HbA_1c_ levels do not equally progress to developing vascular complications [6]. This implies that the pathophysiological basis of these conditions is not solely attributed to glucose. Interestingly, when compared with White individuals with type 2 diabetes, retinopathy and diabetic kidney disease (DKD) appear to be relatively more prevalent in Asian individuals with type 2 diabetes, while cardiovascular diseases occur at a lower prevalence [7]. It is possible that Asian individuals have more severe impairment of insulin secretion, which is reported to be linked to retinopathy, even independently of HbA_1c_ [8]. In support of this, Asian individuals have also demonstrated a better response (measured as a reduction in HbA_1c_) to glucose-lowering drugs, such as incretin mimetics (i.e. glucagon-like peptide 1 [GLP-1] agonists and dipeptidyl peptidase 4 [DPP-4] inhibitors) [9, 10]. However, insulin secretory defects would not explain the excess risks of DKD in Asian individuals, which is suggested to be linked primarily to insulin resistance and obesity imposed by changes in diet [11]. This indicates that pathophysiological processes leading to microvascular complications in type 2 diabetes might operate through glucose-dependent and -independent mechanisms.

Nowadays, the magic of molecular biotechnology to analyse simultaneously millions of genetic variants across the entire genome has opened unprecedented possibilities for genome-wide association studies (GWASs), whole-exome sequencing (WES) and whole-genome sequencing (WGS), and has uncovered multiple common and rare variants associated with diabetes and its related comorbidities [12, 13]. In the coming years, WES and WGS data, together with improved phenotyping, are expected to result in complete genetic mapping and deepen our understanding of causal mechanisms in metabolic diseases.

**Fig. 1:**
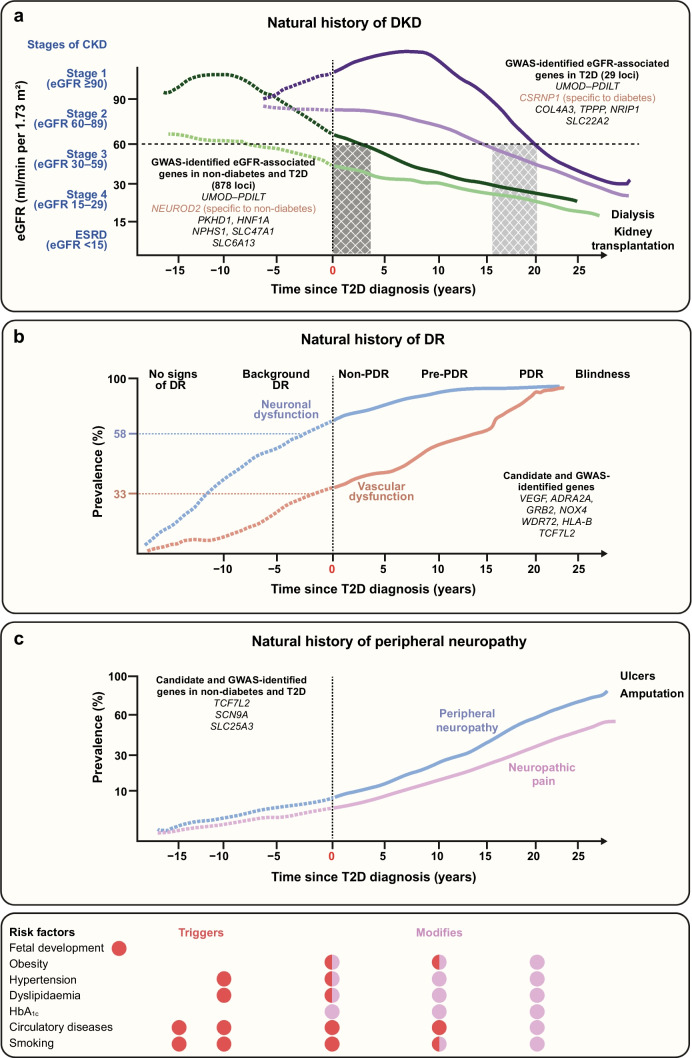
The natural history of the development of DKD, retinopathy and peripheral neuropathy complications in individuals with type 2 diabetes may exhibit different trajectories depending on the time of diabetes onset. Temporal risk factors that may act as triggers of disease (red circles), modifying risk factors (pink circles) or both (half red/half pink circles) are shown, ordered depending on their approximate supposed appearance before or after diabetes onset. (**a**) The different stages of kidney disease (stage 1, normal; stage 2, moderate; stage 3, severe; stage 4; and end-stage renal disease) are defined based on eGFR. The natural history of kidney disease may commence at different blood glucose levels, including both prior to diabetes onset, where blood glucose levels may be normal or elevated, and after diabetes diagnosis (time of diabetes diagnosis indicated by time 0 on the graph). Individuals may experience different kidney function trajectories that either include a stage of hyperfiltration preceding a relatively faster decline in eGFR (dark green line; rapid decline in eGFR illustrated by hatched dark grey area), or a trajectory without hyperfiltration preceding a more moderate–slow pattern of eGFR decline (purple lines; slow decline in eGFR illustrated by hatched light grey area). A decline in kidney function may occur some years before diabetes onset (see green lines) owing to glucose-unrelated aetiologies. In individuals with preceding stage 2 (moderate) CKD, or stage 3 (severe) CKD (defined by a decline in eGFR <60 ml/min per 1.73 m^2^), progression to stage 3 DKD and beyond may occur much faster after the onset of diabetes (green lines) as compared with individuals without a precedent decline in eGFR (purple lines). At the GWAS significance level, 878 genetic loci have been reported as being associated with eGFR in a combined meta-analyses of individuals without and with diabetes [45], while 29 have been found to be associated with eGFR in those with diabetes [38]. Genetic markers for eGFR overlap in individuals with and without type 2 diabetes at different levels of significance, with the same directionality but with different magnitudes of effect. However, *NEUROD2* has been associated with eGFR specifically in individuals without diabetes, while *CSRNP1* has been reported as a specific risk variant in individuals with diabetes. The combination of CKD due to hypertension, obesity and/or other glucose-unrelated factors in individuals with type 2 diabetes may hinder identification of genetic loci specific to DKD. ESRD, end-stage renal disease; T2D, type 2 diabetes. (**b**) The natural history of diabetic retinopathy involves both neuronal (blue line) and vascular (orange line) lesions. Currently, progression to severe pre-proliferative and proliferative stages of diabetic retinopathy are defined only by vascular lesions. However, even prior to diabetes diagnosis (prior to time 0 on the graph), early neurodegenerative processes (blue line) are seen twice as frequently as vascular alterations in individuals without visual signs of diabetic retinopathy (dashed lines). To date, no genetic variants at any appropriate GWAS significance level have been associated with diabetic retinopathy; however, candidate gene studies for severe diabetic retinopathy have validated variants in the *VEGF* and *TCF7L2* genes as being associated with this complication. Notably, the effects of the *TCF7L2* variants are likely to be mediated by subtle or overt hyperglycaemia occurring even prior to diabetes diagnosis. DR, diabetic retinopathy; PDR, proliferative diabetic retinopathy; T2D, type 2 diabetes. (**c**) The natural history of peripheral neuropathy (blue line) and neuropathic pain (pink line) may commence at the normoglycaemic stage, some years prior to diabetes onset (prior to time 0 on the graph, dashed lines). The severity of peripheral neuropathy is often linked with the development of severe neuropathic pain. Most genes associated with these conditions have been identified through candidate gene studies, via combined analysis in individuals with and without diabetes with neuropathy/neuropathic pain. T2D, type 2 diabetes. This figure is available as a downloadable slide

In this review, we discuss progress made in understanding the genetics of microvascular complications, including the necessity of conducting GWASs separately in individuals with type 1 and type 2 diabetes. We emphasise the contribution of different temporal risk factors that might act as triggers of disease early in life (intrauterine and metabolic programming), or late factors accompanying microvascular disease (hyperglycaemia, hypertension, dyslipidaemia and obesity), which need to be taken into consideration in GWASs (see Fig. 1).

## Genetics of diabetes

To date, 568 independent genetic loci have been reported as being associated with type 2 diabetes [13] and around 136 for type 1 diabetes [14]. Cumulatively, many more genetic loci have been discovered for diabetes-related metabolic risk factors [15]. In a large multi-ancestry cohort (*n*=1,178,783 without diabetes, *n*=228,499 with diabetes), a high type 2 diabetes GWAS-derived polygenic risk score (PRS) was shown to be significantly associated with retinopathy, chronic kidney disease (CKD), peripheral artery disease and neuropathy, but not with coronary heart disease or stroke [13]. However, these analyses were performed in the entire cohort without stratification for type 2 diabetes. Thus, the association with microvascular complications could be a corollary to a co-segregation with diabetes. These results, however, clearly motivate further experiments to explain the extent to which aetiopathology and genetic susceptibility to vascular complications are shared between individuals with and without type 2 diabetes. Since the first GWASs for type 2 diabetes, a prominent feature has been the co-segregation of genes involved in cell proliferation, survival and regeneration [12, 13, 16]. The *TCF7L2* gene (a major effector of the canonical Wnt/β-catenin pathway, and the most frequently mutated gene in some cancers, which has also been shown to be important for the degree of malignancy [17]) is hitherto the strongest identified type 2 diabetes susceptibility gene [13]. *TCF7L2* risk variants have also been reported to be associated with all diabetic microvascular complications, including retinopathy [13, 18, 19], nephropathy [20] and neuropathy [21, 22]. Indeed, the association of *TCF7L2* with diabetes and its complications represents robust evidence of biological pleiotropy. Importantly, from the reported evidence of the influence of *TCF7L2* variants on the risk of type 2 diabetes/glucose levels, pancreatic insulin secretion, and the risk of developing retinopathy, nephropathy and/or neuropathy, it cannot be excluded that hyperglycaemia plays a key mediating role underlying the risk of complications. With regard to known type 1 diabetes-associated genetic variants, it is of interest that none of these have been associated with risk of DKD [14].

The interest in the identification of genes contributing to the risk of microvascular complications, particularly kidney disease, stemmed from studies of families and siblings demonstrating high (83%) concordance of kidney disease if probands also had diabetic nephropathy as compared with 17% in siblings whose probands did not have diabetic nephropathy [23]. Familial clustering of DKD has also been reported in individuals with type 1 diabetes [24], as well as in Pima Indian individuals with type 2 diabetes [25]. In these studies, the clustering of DKD could not be accounted for by differences in conventional risk factors, such as diabetes control or elevated blood pressure. However, families share multiple lifestyle factors and exposures besides genes, which are often subtle or unknown and, therefore, not possible to adjust for.

## Genetics of diabetes-associated complications

### Nephropathy

Nephropathy (also referred to as CKD or, in those with diabetes, DKD) is defined by decreased GFR (eGFR) or elevated urinary albumin excretion (albuminuria) [26]. The incidence of DKD is accelerating and it is projected to become the fifth most common cause of death worldwide by 2040 [27]. Very often the decline in kidney function is asymptomatic and individuals might be unaware for a long time that they have kidney disease; therefore, frequent monitoring of eGFR is a prerequisite for early detection of a decline in kidney function. eGFR levels are used to classify different stages of kidney dysfunction, from hyperfunction (hyperfiltration) to normal function and different degrees of reduced function [28]. The prevailing opinion is that the hyperfiltration stage often precedes a decline in kidney function and is associated with increased risk of mortality in both type 1 [29] and type 2 diabetes [30]. Several cross-sectional studies have demonstrated that hyperfiltration is also associated with hypertension, obesity [31], elevated fasting plasma glucose [32] and smoking [33]. Thus, it is plausible that the triggering mechanisms of kidney hyperfunction may be adaptive to protect the body against the excess of toxins or surplus of energy, as in the case with high glucose concentrations. However, in addition to glucose, the kidney also removes other energy substrates (fatty acids and amino acids) and growth factors and, thus, we suggest that prolonged hyperfunction may lead to energetic and/or physiological imbalances, which ultimately result in the initiation of mechanisms to decrease the filtration rate. Metabolic disbalance would result in changes in haemodynamics leading to increased renal pressure, and the presence of hypertension and use of antihypertensive drugs may interfere with the autoregulation of glomerular blood flow [34]. One way to infer causality of an exposure is to use genetic variations that are strongly associated with an exposure as a ‘proxy’ instrument to statistically test and compare the effects and strength of an association directly between the exposure and its genetically determined ‘proxy’ with the risk of a disease, a method called Mendelian randomisation [35]. Mendelian randomisation is based on the biological principle that common genetic variations are randomly assigned at the time of conception and that these are not modifiable, thus providing a robust, static variable as compared with the dynamic exposure variable [35]. Notably, a large Mendelian randomisation analysis recently used genetic variants associated with CKD-related metabolic risk factors at genome-wide significant level to infer their causality in 51,672 individuals without diabetes but with CKD and 958,102 control individuals from the CKD Genetics (CKDGen) Consortium, UK Biobank (UKBB) and the Trøndelag Health Study (HUNT) [36]. Results suggested a causal relationship between several metabolic risk factors and CKD risk, including BMI, systolic blood pressure, lipoprotein(a) levels, HDL-cholesterol (HDL-C), apolipoprotein A-I and type 2 diabetes [36]. Interestingly, in contrast to the reported associations between a type 2 diabetes PRS and DKD, there was little evidence to support the causal effects of a PRS for several glycaemic traits, including levels of fasting insulin, fasting glucose, glucose at 2 h into an oral glucose tolerance test, fasting proinsulin, HbA_1c_, HOMA-B, insulin-like growth factor binding protein 3 and insulin-like growth factor I, on CKD and eGFR in individuals with or without diabetes [36]. However, it cannot be ruled out that these findings, in part, might be related to methodological issues as the number of loci reaching genome-wide significant levels and included in the construction of the PRSs varied greatly between the traits.

To date, several large-scale GWASs, including trans-ethnic meta-analyses, have been reported for kidney function in individuals without diabetes [37, 38] and in individuals with both type 1 and type 2 diabetes [38–40]. Generally, the findings from different studies are not overlapping, partially due to the different definitions used for kidney dysfunction that have been applied to identify cases and controls; however, some reproducible genetic loci have been found. The most consistent genetic variant linked to various definitions of renal traits (eGFR, macroalbuminuria and CKD) was reported in the *UMOD–PDILT* locus in individuals with either type 1 or type 2 diabetes, or both [41]. *UMOD* encodes a uromodulin protein that is ascribed to multifaceted protection from accumulation of salts and infections in the kidney [42]. Moreover, the first large study by the Diabetic Nephropathy Collaborative Research Initiative (DNCRI), performed in more than 19,000 individuals with type 1 diabetes, using a spectrum of ten definitions of DKD based on albuminuria and eGFR, discovered 16 loci at genome-wide significant level across the different definitions [39]. The top signal was a protective common variant (~ 20% allele frequency) in the *COL4A3* gene, which also showed a suggestive association in individuals with type 2 diabetes. The protective effects of this variant have been ascribed to the thinner glomerular basement membrane seen in carriers of the mutant *COL4A3* allele, even in those without any signs of kidney disease. Furthermore, the protective effects were observed specifically in individuals with hyperglycaemia with HbA_1c_ above 58 mmol/mol (7.5%), but not in those with HbA_1c_ less than 58 mmol/mol (7.5%), suggesting a clear modifying effect of hyperglycaemia. Collagens and glycoproteins are major structural components of the mesangial matrix [43]. This makes the *COL4A3* gene a plausible biological link to the well-established pathophysiological and morphological changes in the kidney in diabetes [44]. Thus, a decline in GFR is believed to be associated with an expansion of glomeruli and proximal tubules due to increased extracellular matrix deposition [26]. In this regard, it would be interesting to use a prospective study design to investigate whether the *COL4A3* variant is associated with a slower decline of kidney function. Very recently, the DNCRI researchers expanded their GWAS meta-analyses by combining individuals with type 1 and type 2 diabetes (*n*=27,000) to look for genetic loci associated with eight different definitions of kidney function [40]. The following novel genetic loci were uncovered in the gene-based analyses with no significant heterogeneity between the diabetes types: *COL20A1*, *DCLK1*, *EIF4E*, *PTPRN–RESP18*, *INIP–SNX30*, *LSM14A* and *MFF*. These findings further support the role of collagens and highlight the importance of mitochondria in all DKDs. An integrative analysis of GWAS with tubular and glomerular transcriptomics allowed for the discovery of two more loci, *TENM2* and *SNX30*. In line with a previous report [36], Mendelian randomisation analyses supported a causal effect of BMI on the risk of DKD; however, this approach could not find any suggestive evidence for the causal effects of other previously reported metabolic traits, including type 2 diabetes and HDL-C [40]. A very important observation was a lack of replication of the discovered genetic loci across the diabetes types [40], suggesting insufficiently powered studies, or differences in the underlying biology and molecular causes of kidney damage in individuals with different diabetes subtypes.

The observed differences between genetic susceptibility to kidney dysfunction in individuals with type 1 and type 2 diabetes, in combination with the perception of shared genetic predisposition to kidney dysfunction in individuals without diabetes and with type 2 diabetes, may not only point to the importance of conducting GWAS analyses separately in individuals with different diabetes types, but also the importance of understanding the contribution of potential genetic (type 1 or type 2 diabetes-established loci) and non-genetic (HbA_1c_, age at onset, blood pressure, dyslipidaemia, comorbidities) confounders. Type 1 diabetes is an autoimmune disease, while type 2 diabetes is a more environmentally triggered metabolic disease [3], where changes in gene expression are, to a great extent, altered by epigenetic modifications. Interestingly, recent results from integrative GWAS meta-analyses for eGFR (using data from the CKDGen Consortium, UKBB, Million Veteran Program, Population Architecture using Genomics and Epidemiology [PAGE] study and SUMMIT study) with epigenomic and transcriptomics, demonstrated larger heritability estimates for methylation quantitative trait loci (meQTL) than for expression quantitative trait loci (eQTL) [45]. From the combined analyses of data from GWAS for kidney dysfunction, meQTL and gene eQTL, the authors comprehensively mapped 878 eGFR-associated loci. Creating a prioritisation score based on eight omics phenotypes or analytical approaches (eGFR, kidney gene expression and methylation, GWAS variant co-localisation analyses using data from GWAS, meQTL and eQTL, open chromatin areas, single-cell chromatic accessibility analyses and Mendelian randomisation) lead to identification of the rs2252281 variant in the *SLC47A1* gene (encoding solute carrier family 47 member 1 [SLC47A1]) as one of the top prioritised signal, with the strongest effect size on eGFR (*p*=10^−75^), CpG methylation (*p*=10^−39^) and *SLC47A1* expression (*p*=10^−8^), and co-localisation of all three of these traits (Bayesian posterior probability=0.98) [45]. Subsequent functional mice studies confirmed the causal role of Slc47a1 in mediating low-dose-cisplatin-induced kidney injury [45]. SLC47A1 is also known as multidrug and toxin extrusion protein 1 (MATE1), which is a cation transporter. Carriers of genetic variants in *SLC47A1* have demonstrated a better response to metformin therapy than non-carriers due to impaired excretion of metformin in urine and, therefore, increased concentrations in the blood [46]. While retention of metformin might have overall beneficial effects, impaired renal clearance may cause accumulation of other drugs and toxins, such as cisplatin, in the proximal tubular cells and, thereby, induce nephrotoxicity [47]. In an extensive post-GWAS annotation analysis, the researchers highlighted the key contribution of proximal tubules, metabolism and cell-death pathways in kidney function [45].

Recently, members of the CKDGen Consortium performed a large comparative GWAS analysis in individuals without and with diabetes (*n*=1,296,113 without diabetes, *n*=178,691 with diabetes, not separated by diabetes type) for eGFR and/or CKD [38]. Notably, in addition to the above-mentioned *SLC47A1* gene*,* these analyses identified other salt-forming organic cation transporters, namely *SCL22A2* and *SLC34A1* genes, as being linked to kidney function in all diabetes types. Interestingly, associations of eGFR with PRS based on 634 GWAS-identified variants (previously identified by the CKDGen Consortium using a general population of European ancestry [37]) and seven diabetes-related variants (previously identified by Winkler et al [38]) were strong and directionally consistent in individuals with and without diabetes. This may suggest that hyperglycaemia has a modifying rather than causal role in DKD in adults (the age of participants in the different cohorts was between 40 and 70 years) or that a significant proportion of individuals with diabetes do not have DKD.

### Retinopathy

Almost all individuals with diabetes have some degree of retinal and/or visual impairment, making retinopathy a very common complication of diabetes. Diabetic retinopathy is a neurodegenerative complication involving both vascular and neuronal units of the retina [48, 49]. At present, the degree of diabetic retinopathy progression is defined as non-proliferative, pre-proliferative and proliferative, but this is only based on signs of vascular lesions in the retina [50]. However, in the European Consortium for the Early Treatment of Diabetic Retinopathy (EUROCONDOR) study, using multifocal electroretinogram and spectral domain optical coherence tomography, 58% of individuals with type 2 diabetes, with a disease duration of at least 5 years and no apparent fundus abnormalities showed signs of neurodegeneration, and only 33% had vascular alterations without neurodysfunction [51]. This demonstrates that neurodegeneration occurs in individuals with no visible signs of diabetic retinopathy, whereby thinning of the nerve fibre and ganglion cell layers occur without changes in the vasculature. Current treatments for diabetic retinopathy, including anti-vascular endothelial growth factor (VEGF) and laser photocoagulation, target the late proliferative stages of diabetic retinopathy and aim to reduce the vascular alterations seen at funduscopic examination [49]. However, only half of individuals having anti-VEGF treatment benefit from it, which indicates the existence of other, perhaps non-vascular-related, factors that underly the pathological changes associated with proliferative diabetic retinopathy [49]. This further indicates that evaluation of diabetic retinopathy should also include assessment of the function of the neuronal retina. It is well established that diabetic retinopathy is linked to other complications of diabetes such as neuropathy.

Multiple candidate gene studies have suggested several loci that are associated with diabetic retinopathy including *VEGFA, AKR1B1, AGER, ICAM1* and *MTHFR* [52–55]. The strongest supportive evidence for a role in the pathogenesis of severe forms of diabetic retinopathy has been provided for the *VEGF* gene [55]. To date, several GWASs using different populations and different definitions of diabetic retinopathy have been reported [56]. However, none of the results were consistently replicated, most likely due to insufficient sample sizes and the inconsistent definitions of diabetic retinopathy used in the very early candidate gene studies. Nevertheless, it is worth mentioning that among several biologically interesting signals, a variant in the *NOX4* gene, was found to be associated with diabetic retinopathy in the Genetics of Diabetes Audit and Research in Tayside and Scotland (GoDARTS) study [57]. NADPH oxidase (NOX)4 is a member of the NOX family of enzymes that function as catalytic subunits of the mitochondrial NOX complex. NOX4 has also been suggested to be a major source of oxidative stress in the failing heart [58]. Is it thus possible to speculate that a lack of replication of these findings in some of the other study cohorts (the Australian Diabetic Retinopathy Genetics Case–Control study, the Cardiovascular Health Study 2 [CHS2], the Finnish Diabetic Nephropathy Study [FinnDiane], the Genetics of Kidneys in Diabetes study [GoKinD] and the Epidemiology of Diabetes Interventions and Complications [EDIC]), as assessed in a meta-analyses for this variant [57], might, in part, be explained by confounding effects related to an overrepresentation of underlying cardiovascular pathologies in the rather elderly GoDARTS population? Another interesting genome-wide significant signal linked to sight-threatening retinopathy has been identified in the vicinity of the *GRB2* gene (encoding growth factor receptor bound protein 2) in a meta-analysis combining White and Asian populations with type 1 and type 2 diabetes [59]. This gene encodes an epidermal growth factor receptor-binding protein that is expressed in mouse and human retinas, and elevated expression of this gene was linked to neovascular retinopathy in a mouse model of retinal stress [59, 60]. *GRB2* is also involved in VEGF signalling, which additionally supports its biological relevance in relation to diabetic retinopathy [61].

In a smaller study of populations in Ghana and Nigeria in West Africa, GWAS was performed applying the ‘extreme phenotype’ approach (‘super’ controls, *n*=227; selected cases with altered neovascularisation and or retinal detachment, *n*=64) [62]. Out of four genome-wide significant loci identified, one locus in the protein-coding *WDR72* gene was replicated in African Americans [63]. Tryptophan‐aspartate repeat domain 72 (WDR72) is highly expressed in the retina and kidney epithelium, and *WDR72* variants were associated with changes in HbA_1c_ in the intensive arm of the DCTT [64], making this gene a promising biological candidate for the risk of progression of diabetic retinopathy. Other variants with genome-wide significance were located in the *HLA-B, GAP43* and *AL713866* genes [64]. Notably these genes are expressed in the retina and in the pancreas, which might further support the epidemiological evidence of a link between elevated risk of retinopathy in individuals with insulin-deficient type 2 diabetes [65]. So far, a rather limited number of WES studies for diabetic retinopathy that use the ‘extreme phenotype’ approach are reported [66, 67]. A WES study for diabetic retinopathy in African American individuals (*n*=70) identified 44 genes (19 of them novel) that reached a genome-wide significance level including variants in the *VEGFB* gene [67].

Candidate gene approaches have confirmed a role for VEGF as a marker of vascular pathology in the progression of diabetic retinopathy [55]. This, however, could in part be related to the current definition of retinopathy, which is staged based on the severity of vascular alterations [50]. However, at present, the definitions of the different stages of the natural history of diabetic retinopathy lack evaluation of neurodegenerative processes. Our recent candidate gene studies, including established type 2 diabetes loci, highlighted variants in *ADRA2A* (encoding adrenergic receptor 2 A), and also in *PROX1* (encoding a stem cell progenitor marker) and *PCSK9* (encoding an LDL receptor-degrading enzyme), in famine-linked risk of proliferative diabetic retinopathy [68]. These genes are all expressed in neurons and neuroretinal glial cells, supporting the role of neurodegeneration in the pathogenesis of diabetic retinopathy.

### Neuropathy

Neuropathy affects 30–60% of individuals with diabetes depending on the duration of the disease [69]. Damage or lesions of neuronal fibres have multiple aetiologies, and the definition of neuropathy comprises various pathologies with peripheral and autonomic neuropathy being the major types. Clinical presentation of nerve-fibre deterioration can vary from asymptomatic to neuropathic pain [70]. Metabolic risk factors, such as dyslipidaemia and altered sphingolipid metabolism, are suggested to be involved in nerve injury, in addition to traditional risk factors, such as hyperglycaemia and obesity [70]. Neuropathic pain is also linked to different underlying conditions of which diseases of the circulatory system and osteoarthritis are the most common [71]. Assessment of neuropathic lesions is difficult in the clinic due to difficulties in measuring nerve degeneration accurately and precisely, particularly at the early stages. Diabetic neuropathy causes development of ulcers and might lead to amputation of the lower limbs.

To date, there are only a few GWASs published for diabetic neuropathy and neuropathic pain [72, 73]. A recent literature review of 29 articles highlighted a number of genes as being associated with diabetic neuropathy and/or neuropathic pain; these genes were involved in ion channel activity (*SCN9A*, *SLC6A4*, *CACNG2*), neurotransmission (*OPMR1*, *COMT*, *PRKCA*, *MPZ*), immune response (*HLA*, *IL6*, *TNF-α*) and metabolism (*TF*, *CP*, *GCH1*, *TFRC*, *SLC11A2*) [74]. Among the candidate genes researched, variants in *GCH1* [75], *KCNS1* [76] and *P2X7* [77] have been linked to neuropathic pain persistence/severity, but much of the literature in this area (particularly in diabetes) has suffered from modest sample sizes, inconsistent phenotyping and limited replication. There has been progress in discovering causal mutations in ion channels for a number of primary non-diabetes-associated neuropathic pain conditions [78–80]. For example, rare mutations in the *SCN9A* gene (encoding the voltage gated sodium channel Na_V_1.7) have been linked to several chronic pain disorders, and mutations in *TRPA1* (encoding a transient receptor potential [TRP] channel) cause familial episodic pain syndrome [80]. Familial primary neuropathic-pain conditions are rare but provide mechanistic insight into the pathogenesis of neuropathic pain [78, 79]. Recently GWAS analyses using UKBB data identified a genome-wide significant signal in the mitochondrial phosphate carrier gene *SLC25A3* which was associated with neuropathic pain, as defined by use of anti-neuropathic prescription drugs [72]. There are also currently ongoing GWAS and WES efforts to discover genes associated with diabetic neuropathic pain in the European Union-funded DOLORisk Consortium [81].

GWASs of microvascular complications in individuals with type 1 or type 2 diabetes have, so far, identified only a small number of genes at genome-wide significance level [38–41, 56, 57, 59, 62, 72]. Apart from the modest sample sizes in the GWAS analyses for microvascular complications (compared with GWASs for type 1 or type 2 diabetes), consideration of the co-existence of metabolic risk factors (HbA_1c_, obesity, hypertension, dyslipidaemia, and treatments for diabetes and comorbidities) is of great importance. However, regulation of these dynamic metabolic derangements can be modified by a range of temporal environmental factors, which play distinct roles during the course of life and disease(s) [82]. One relevant factor may be the role of early metabolic programming events [83]; these might lead to priming of vasculature and nerve tissue vulnerability, as well as hypertension, which later manifests pathologically in a hyperglycaemic environment.

During recent decades, emerging evidence suggests a significant contribution of perinatal factors in the predisposition to several diseases and disease manifestations later in life [84]. Thus, intrauterine nutritional deprivation and persistent psychological stress during pregnancies have been linked to increased risks of abnormal glucose tolerance, hypertension [85], cardiovascular diseases [86], kidney dysfunction and type 2 diabetes [87]. The underlying mechanisms might involve epigenetic changes that occur during fetal life, including in immature stem cells, which may permanently change key cellular functions in all affected organs in type 2 diabetes throughout life [88]. In support of the fetal programming hypothesis in the predisposition to vascular complications of diabetes, we have recently reported a disproportionally elevated risk for proliferative diabetic retinopathy in individuals with type 2 diabetes: in two independent populations (from Ukraine and Hong Kong), we found that the incidence of proliferative diabetic retinopathy was disproportionately higher in individuals who experienced perinatal exposure to famine [89]. Furthermore, our experimental data on starved-for-glucose embryonic retinal cells allowed us to generate a hypothesis of irreversible and detrimental reprogramming of formation of the entire neurovascular unit during retinal development after early-life exposure to glucose starvation [90]. Specifically, using transcriptomic analysis, we found a profound decrease in the expression of neuronal markers, while genes encoding for vascular markers were upregulated, similar to the diabetes-associated increase in angiogenesis in diabetic retinopathy [90].

## Mechanisms underlying diabetes complications illuminated by genomic studies: glycolysis, mitochondrial function and DNA damage

Although GWASs for diabetes complications are beginning to identify a number of associated genetic loci, the underlying biology remains to be understood. To this end, our recent genomics findings in individuals with long-term type 1 diabetes support a significant role for genetic regulation of processes involved in glycolysis, mitochondrial dysfunction, cell regeneration and DNA damage [91]. Specifically, in individuals who were largely free from major macro- or microvascular complications despite more than 30 years of diabetes duration (non-progressors), we found a synergistic reduction in expression of mitochondrial oxidative phosphorylation (OXPHOS) genes in blood as compared with individuals with microvascular diseases (rapid progressors). This reduction was correlated with reduced expression of genes encoding DNA repair enzymes in the base excision repair pathway. This transcriptomic pattern positively correlated with higher insulin sensitivity and lower liver fat indices [91]. During the last decade, emerging evidence supports the notion that increased intracellular energy overload due to chronic hyperglycaemia results in overproduction of superoxide in the mitochondria, elevated oxidative stress and increased risk of vascular complications [92]. In line with these notions, our findings indicate that modest downregulation of mitochondrial OXPHOS may be beneficial in preserving the physiological functions of reactive oxygen species (ROS) as a signalling molecule in non-progressors, thereby contributing to organ protection from oxidative stress. Synergistic downregulation of genes encoding DNA repair enzymes may also reduce the genotoxic effects of ROS, reducing DNA damage. Metabolomic profiling suggested that non-progressors have lower levels of pyruvate (a glycolytic metabolite), thiamine monophosphate (a pentose phosphate pathway cofactor) and erythritol (a pentose phosphate pathway intermediate product), as well as higher levels of phenylalanine, glycine and serine [93]. Cumulatively, these findings allow us to hypothesise that glycolytic substrates may be beneficially shunted towards the pentose phosphate and one-carbon metabolism pathways to promote nucleotide biosynthesis in the liver [93]. This would be in line with more effective repair processes and cell renewal and, thereby, reduced the propensity to cell and organ damage caused by chronic hyperglycaemia or oxidative stress.

## Biology vs methodology

The genetic architecture of the risk of complications seems to be different in individuals with autoimmune type 1 diabetes and non-autoimmune type 2 diabetes. The modifying mechanisms might include differential pathophysiological mechanisms. We need to adopt or design methodologies that will determine the exact importance of contributory factors, including glucose, obesity, hypertension and the fetal environment, and biological pleiotropy, which together might increase the power of detecting genetic drivers of various complications in individuals with different diabetes types. Optimally, as part of prospective studies with repeated clinical measurements, GWASs should be conducted to shed light on the molecular mechanisms underlying disease and to elucidate how diseases should be staged, taking into account the underlying natural history of damage to a specific organ. In Fig. 1, we have tried to map the different processes in the development of dysfunction in the kidneys, eyes and nerves. Notably, largely common genetic factors for eGFR in the general population and individuals with type 2 diabetes [38] might indicate the presence of a significant proportion of kidney dysfunction that is not related to glucose in individuals with diabetes. Several studies have demonstrated that neurodegeneration is an early process in the pathophysiology of progression of diabetic retinopathy [49, 51]; however, current guidelines in the clinic are still only focused on evaluating vascular lesions [50]. Accumulating evidence suggests that corneal confocal microscopy for detection of damage of small neuronal fibres can be a useful early screening tool for peripheral neuropathy [94]. The severity of peripheral neuropathy is often linked with the development of neuropathic pain [95]. Altogether, these points emphasise the necessity of better and deeper phenotyping of complication status in diabetes for genetic analyses that may enable us to determine specific mechanisms underlying these complications and also to illuminate the role of different aetiopathologies in the development of diabetes-related complications and comorbidities.

## Conclusions

Dissecting the genetic determinants of diabetes complications is a complex endeavour that requires understanding of molecular mechanisms preceding diabetes onset, as well as triggers in early life and modifying risk factors in later life. Disentangling the genetics of diabetes complications relies on precise definitions of corresponding organ damage, reflecting the natural history of the complication in question. Although hyperglycaemia is a strong driver of complication development, accumulating evidence suggests that other factors either act in synergy with hyperglycaemia or counteract it in a way that protects some individuals [93]. We demonstrated that intrauterine programming during early fetal developmental may play an important role in the development of proliferative diabetic retinopathy by making vessels and nerves more vulnerable and, thereby, susceptible to the damaging effects of chronically elevated glucose levels [68, 89, 90]. However, the relative contribution of low insulin or high glucose levels to neuron damage and related risks of retinopathy and neuropathy still needs to be determined. On the other hand, recent findings from studies on novel subtypes of adult diabetes suggested that insulin-resistance mechanisms rather than glucose-dependent pathways may significantly impact predisposition to kidney dysfunction [65], thus more data may be beneficial to support this association. Mitochondrial function and DNA repair/damage processes appear to be a common denominator for complications in diabetes; however, although many correctly hypothesised that these processes would promote complications by impacting defective oxidative metabolism, in contrast to what many believed, they can also be protective [96]. Thus, genetically determined reduced generation of ROS might be protective in situations of chronically elevated glucose by diminishing damaging effects while preserving favourable biological functions of ROS signalling. Understanding how different peripheral organs shift and utilise energy is critical to understanding the pathophysiology of glucose-related and -unrelated tissue and organ damage. It is important to know whether adjusting GWAS analyses for genetic (diabetes-type loci) and non-genetic (glycaemic exposure/HbA_1c_) factors, along with factors related to the fetal environment is relevant for unravelling a larger number of genetic loci associated with diabetes-related complications. Multi-trait phenotype GWASs to address antagonistic or synergistic pleiotropy of genetic effects may help to illuminate the link between different complications (co-)occurring in individuals with diabetes. Access to the large and well-characterised cohorts with WES and WGS data, along with multi-omics data integration (proteomics, transcriptomics) will provide information about how genetic variants may regulate and modify the function of different organs. Although not yet fully applicable in the clinic, emerging artificial intelligence and integrative machine learning tools might hold great potential. These tools, combined with profiling of clinical and genetic risk for diabetes and associated complications, might aid in the future design of clinical decision support systems to personalise treatment of diabetes and its complications.

## Supplementary Information

Below is the link to the electronic supplementary material.

Supplementary file1 (PPTX 166 KB)

## Abbreviations

CKD: Chronic kidney disease
CKDGen: Chronic Kidney Disease Genetics (Consortium)
DKD: Diabetic kidney disease
DNCRI: Diabetic Nephropathy Collaborative Research Initiative
eQTL: Expression quantitative trait loci
GoDARTS: Genetics of Diabetes Audit and Research in Tayside and Scotland
GWAS: Genome-wide association study
HDL-C: HDL-cholesterol
meQTL: Methylation quantitative trait loci
NOX: NADPH oxidase
OXPHOS: Oxidative phosphorylation
PRS: Polygenic risk score
ROS: Reactive oxygen species
SLC47A1: Solute carrier family 47 member 1
UKBB: UK Biobank
VEGF: Vascular endothelial growth factor
WES: Whole-exome sequencing
WGS: Whole-genome sequencing
