# Supplementary material for: Genetics of diabetes-associated microvascular complications
Source: Diabetologia. 2023 Jul 14;66(9):1601–13. doi: 10.1007/s00125-023-05964-x (PMC10390394; doi:10.1007/s00125-023-05964-x)
Supplement: Supplementary file 1 — Supplementary file1 (PPTX 166 KB) [file 125_2023_5964_MOESM1_ESM.pptx]

## Slide 1
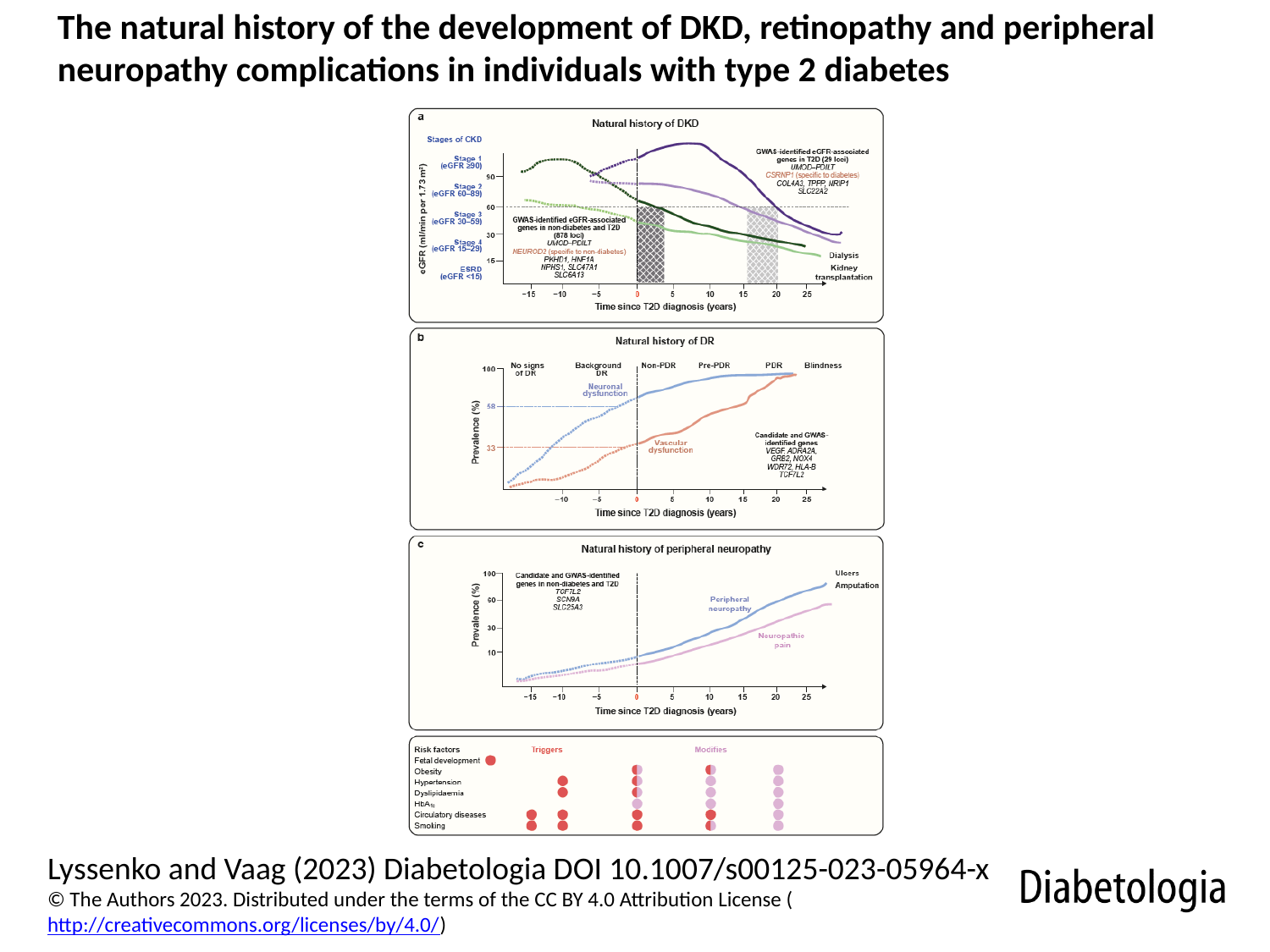

The natural history of the development of DKD, retinopathy and peripheral neuropathy complications in individuals with type 2 diabetes
Lyssenko and Vaag (2023) Diabetologia DOI 10.1007/s00125-023-05964-x
© The Authors 2023. Distributed under the terms of the CC BY 4.0 Attribution License (http://creativecommons.org/licenses/by/4.0/)
